# Overcoming Resistance to Anti-EGFR Therapies: Mechanisms of Cetuximab and Panitumumab Resistance and Emerging Combination Strategies

**DOI:** 10.3390/ph19071041

**Published:** 2026-07-03

**Authors:** Gabriela Henrykowska, Dorota Bartusik-Aebisher, Klaudia Dynarowicz, Tamil Selvan Ramesh, Barbara Smolak, David Aebisher

**Affiliations:** 1 Department of Epidemiology and Public Health, Faculty of Medicine, Medical University of Lodz, T. Kosciuszki 4, 90-419 Lodz, Poland; 2 Department of Biochemistry and General Chemistry, Faculty of Medicine, University of Rzeszów, 35-310 Rzeszów, Poland; dbartusikaebisher@ur.edu.pl (D.B.-A.); kdynarowicz@ur.edu.pl (K.D.); 3 Medical Science Club, Faculty of Medicine, University of Rzeszów, 35-310 Rzeszów, Poland; tr131820@stud.ur.edu.pl; 4 Department of Diagnostic Imaging and Nuclear Medicine, Faculty of Medicine, University of Rzeszów, 35-310 Rzeszów, Poland; bsmolak@ur.edu.pl; 5 Department of Photomedicine and Physical Chemistry, Faculty of Medicine, University of Rzeszów, 35-959 Rzeszów, Poland

**Keywords:** anti-EGFR, cetuximab, panitumumab, intrinsic resistance, acquired resistance, colorectal cancer, MAPK pathway, PI3K-AKT-mTOR axis, combination strategies, ctDNA

## Abstract

Cetuximab and panitumumab are anti-EGFR monoclonal antibodies widely used for the treatment of colorectal cancers. However, due to various mechanisms of resistance to these targeted therapies, the patients’ responses vary. These resistances remain a major obstacle in treatment and overcoming them has become a key emphasis of current therapeutic strategies. Intrinsic and acquired resistance often lead to reactivation of downstream signaling pathways, mainly the RAS-RAF-MEK-ERK (MAPK pathway) and PI3K-AKT axes. Prior existing mutations in *KRAS*, *NRAS*, and *BRAF* result in primary resistance by constantly activating the signals, irrespective of EGFR inhibition. That said, acquired resistance manifests under therapeutic burden through the process of clonal evolution via *KRAS* and *BRAF* alterations, restoring MAPK pathway activity despite EGFR inhibition. In addition to those mutations, tumor cells exploit mechanisms independent of EGFR, such as the pathway bypass, which includes amplification of ERBB family receptors like HER2 (ERBB2) and activation of MET signaling. To overcome these resistances, novel strategies have emerged, which target multiple nodes within the oncogenic networks. Such methods include vertical pathway inhibition, multi-kinase inhibition, liquid-biopsy-guided therapy, and anti-EGFR rechallenge. Reactivation driven by secondary mutation can be prevented by targeting multiple nodes within the MAPK cascade simultaneously, which is referred to as the vertical pathway inhibition. Overall, this review underscores that overcoming therapeutic resistance requires a multidimensional approach that integrates molecular profiling, rational combination therapies, and adaptive treatment. Finally, these advances underscore the shift toward precision oncology, where therapy is tailored to tumor evolution, leading to improved response and patient outcome.

## 1. Introduction

Colorectal cancers (CRCs) are the third most common malignancy and the second leading cause of cancer death worldwide, with the highest incidence in areas including East Asia, North America, and Europe [1,2]. Treatment of metastatic CRC (mCRC) includes monoclonal-antibody-targeted therapy, such as anti-epidermal growth factor receptor (anti-EGFR) and anti-vascular endothelial growth factor (anti-VEGF) [3,4,5]. However, the efficacy of anti-EGFR therapy is limited by a complex spectrum of resistance mechanisms. These include primary (intrinsic) resistance, in which tumors exhibit preexisting molecular alterations that prevent EGFR blockade, and acquired resistance, which develops during treatment through clonal evolution and selection of resistant subpopulations. Current evidence indicates that genomic alterations, particularly mutations in the RAS, BRAF, and PIK3CA genes, as well as acquired alterations involving EGFR, HER2, and MET, are the dominant and best-characterized resistance factors. In contrast, nongenetic mechanisms, including tumor-microenvironment-dependent signaling, epithelial-to-mesenchymal transition, and growth-factor-dependent bypass pathways, are increasingly recognized as important modulators of therapeutic response. Although their relative contribution is not clearly defined, these processes may interact with genomic alterations and contribute to treatment failure in tumors in which genetic resistance factors cannot be identified. EGFR antagonists, such as cetuximab (a chimeric immunoglobulin G1/IgG1 monoclonal antibody) and panitumumab (a human immunoglobulin G2 kappa monoclonal antibody), are used to treat patients with RAS wild-type tumors. They prevent ligand binding and receptor dimerization, indirectly inhibiting kinase activation by binding to the extracellular domain/exodomain of EGFR. This process prevents autophosphorylation and blocks downstream signals, thereby stopping tumor proliferation and survival [6,7].

EGFR is a prototypical transmembrane receptor tyrosine kinase (RTK) belonging to the ErbB family [8]. RTKs are high-affinity cell surface receptors that mediate signal transduction upon ligand binding. After ligand binding, the EGFR undergoes dimerization, activating its intracellular kinase domain and initiating the downstream signaling cascades. The RAS-RAF-MEK-ERK and PI3K-AKT are intracellular downstream signaling pathways that are responsible for the regulation of cell proliferation, differentiation, apoptosis, and angiogenesis. Such critical pathways are activated due to receptor dimerization after conformational change of EGFR, hence making EGFR a great target in therapeutic oncology. However, its clinical outcome is greatly affected by tumor resistance. Patients who fail to respond to therapy initially exhibit intrinsic resistance, whereas those who relapse after initial response are associated with acquired resistance [9]. Notably, a subset of patients with *RAS* wild-type tumors still fail to respond to anti-EGFR therapy, showcasing the complexity of resistance.

Mutational status of downstream signaling molecules is one of the crucial determinants of anti-EGFR therapeutic response. Mutations in *KRAS* and *NRAS* lead to continuous activation of the MAPK pathway, which makes the EGFR inhibition ineffective. Such mutations represent around forty percent of mCRC and are greatly linked to cetuximab and panitumumab resistance. In addition, mutations in *BRAF V600E* are associated with poor prognosis and low sensitivity to anti-EGFR monotherapy; however, this differs when combined with targeted inhibitors, highlighting the importance of novel combination therapies [10].

Along with mutations in downstream signaling molecules, mechanisms such as parallel receptor tyrosine kinase activation lead to therapeutic resistance. Amplification of HER2 or MET can bypass EGFR inhibition, which highlights tumor adaptation and stresses the importance of combination therapy. Patients who initially respond to anti-EGFR develop acquired resistance within 12 to 18 months due to secondary mutations in *KRAS* genes, EGFR exodomain alteration, and clonal selection [9]. This tumor evolution highlights the importance of longitudinal molecular monitoring.

These challenges necessitate the need to understand molecular mechanisms underlying resistance, which is again crucial for optimizing therapeutic strategies. With advances in genomic profiling, liquid biopsy has greatly improved the identification of resistance drivers and contributed to development of novel combination strategies that target multiple pathways simultaneously [4,5].

This review provides a comprehensive overview of such mechanisms leading to anti-EGFR resistance, with a particular focus on cetuximab and panitumumab. It will also explore critical genetic alterations, including *KRAS*, *BRAF*, *HER2*, and *MET,* along with emerging strategies to overcome resistance, ongoing clinical trials, and future directions in precision oncology.

## 2. Materials and Methods

**Figure 1:**
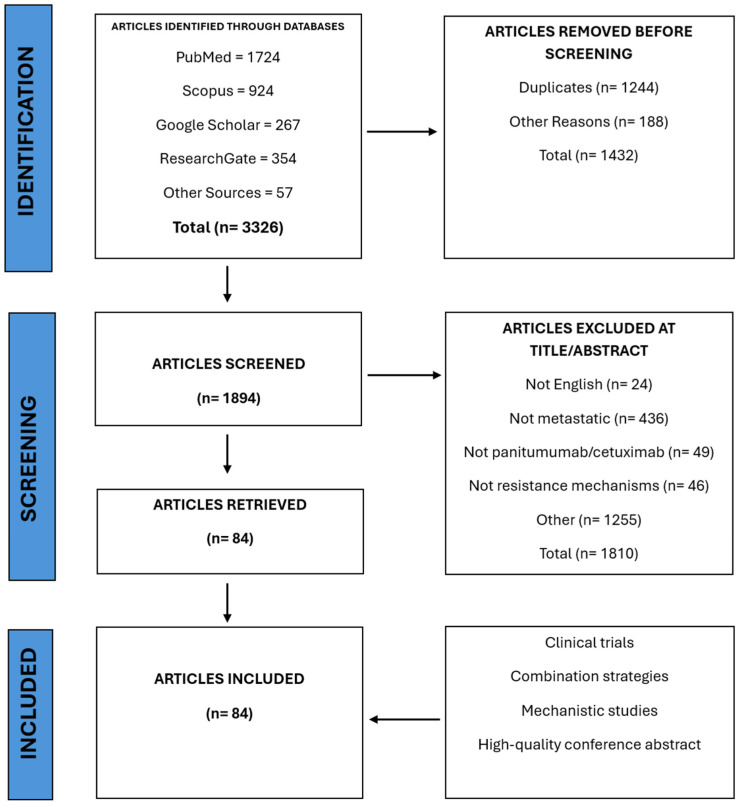
PRISMA flow diagram of the included studies.

A thorough literature search was conducted from 15 April to 20 May 2026 using electronic medical databases and supplementary sources, such as PubMed, Scopus, Google Scholar, Research Gate, and other sources. Additional records were also retrieved from health websites, conference abstracts, and hand searching reference lists of included reviews and key original articles. The following search string was used in each database: (1) cetuximab/panitumumab, and/or (2) colorectal cancers/metastatic colorectal cancer, and/or (3) drug resistance/resistance mechanisms/therapeutic failure, and/or (4) combination therapy/novel strategies. Database-specific syntax was adapted when necessary. Inclusion criteria were peer-reviewed original research, clinical trials, experimental studies, reviews, and high-quality conference abstracts. Articles published in English within the last 25 years (published between 2001 and May 2026), studies focusing on metastatic colorectal cancer treated with cetuximab or panitumumab, studies addressing resistance mechanisms (both intrinsic and acquired), combination therapy strategies, along with recent therapeutic advances, were given priority. Exclusion criteria included non-English publications, studies on non-metastatic or early-stage CRCs, studies not involving cetuximab or panitumumab, studies not addressing resistance mechanisms or combination strategies, duplicate records, and studies with unavailable full text due to institutional access restrictions. A key limitation of the study was restricted institutional access to certain journals and databases, which may have limited retrieval of some full-text articles. We followed the PRISMA 2020 flow diagram (Figure 1) for the study selection process for the review on resistance mechanisms and emerging combination strategies in cetuximab- or panitumumab-treated mCRC. A total of 3326 records were identified from database searches (PubMed = 1724, Scopus = 924, Google Scholar = 267, Research Gate = 354, and other sources = 57). After removal of duplicates (n = 1244) and records published before 2001/other reasons (n = 188), a total of 1894 records proceeded to title and abstract screening. At this stage, 1810 records were excluded for the following reasons: not English (n = 24), not metastatic (n = 436), not involving cetuximab or panitumumab (n = 49), not focusing on resistance mechanisms (n = 46), and other reasons (n = 1255). The remaining 84 full-text records were retrieved, and all met eligibility criteria for inclusion. Included studies comprised clinical trials, combination strategies, mechanistic studies, and high-quality conference abstracts. All the figures and tables included in the manuscript were created and hand-drawn by the authors using software tools such as Microsoft Word version 2602 and GoodNotes version 5.

## 3. EGFR-Mediated RAS-RAF-MEK-ERK and PI3K-AKT-mTOR Signaling

In CRCs, EGFR functions as a central regulator of signaling on the cell surface; when triggered, it activates two primary intracellular pathways, namely, the MAPK and PI3K/AKT pathways.

### 3.1. The MAPK Pathway (RAS-RAF-MEK-ERK)

It is a primary pro-growth signaling cascade that translates signals from the cell surface to the nucleus to trigger cell division.

Ligands such as the EGF bind to EGFRs, which are located on the cell membrane, resulting in dimerization and activation of the EGFR. The activated EGFR recruits adapter proteins that convert the RAS to an active form. The resulting active RAS recruits RAF (a MAP3K) to the cell membrane. Furthermore, the RAF phosphorylates MEK (a MAP2K), which later, in turn, phosphorylates ERK (the MAPK). The ERK then translocates into the nucleus, where it activates MYC and FOS (transcription factors), ultimately driving the cell cycle forward [10,11].

### 3.2. The PI3K-AKT-mTOR Axis

**Figure 2:**
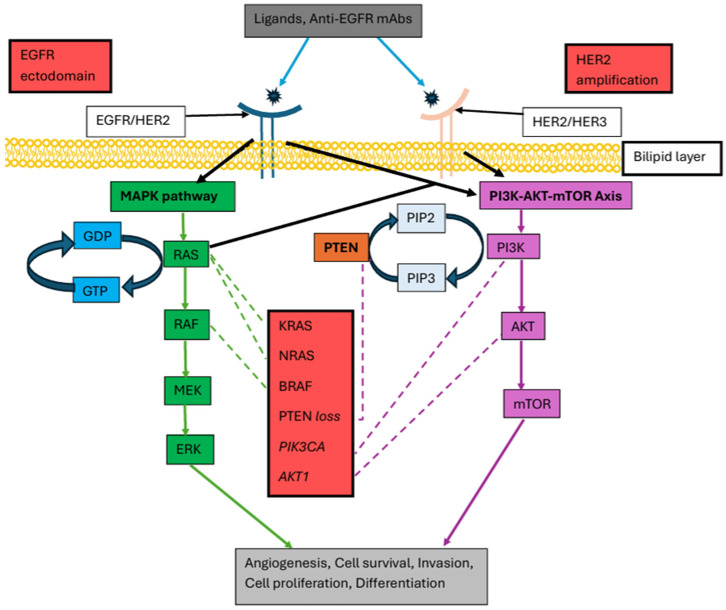
Schematic representation of EGFR signaling pathways and mechanisms of resistance to anti-EGFR monoclonal antibodies (cetuximab and panitumumab). Ligand-bound EGFR activates two main downstream cascades: the MAPK pathway (RAS-RAF-MEK-ERK) and the PI3K-AKT-mTOR axis (via PIP2/PIP3). Anti-EGFR monoclonal antibodies block receptor activation. Resistance arises through mutations in KRAS, NRAS, BRAF, PIK3CA, EGFR ectodomain, PTEN loss, and HER2 amplification, leading to uncontrolled proliferation, survival, and invasion. Green boxes: MAPK pathway, purple boxes: PI3K-AKT-mTOR axis, and red boxes: resistance mechanisms and mutations. Abbreviations: AKT—protein kinase B (PKB); BRAF-B—rapidly accelerated fibrosarcoma; EGFR—epidermal growth factor receptor; ERK—extracellular signal-regulated kinase; GDP—guanosine diphosphate; GTP—guanosine triphosphate; HER2—human epidermal growth factor receptor 2; HER3—human epidermal growth factor receptor 3; KRAS—Kirsten rat sarcoma virus; MAPK—mitogen-activated protein kinase; MEK—mitogen-activated protein kinase kinase; mTOR—mammalian target of rapamycin; NRAS—neuroblastoma rat sarcoma virus; PI3K—phosphoinositide 3-kinase; PIK3CA—phosphatidylinositol 4,5 bisphosphate 3 kinase catalytic subunit alpha; PIP—phosphatidylinositol 4,5 bisphosphate; PIP3—phosphatidylinositol (3,4,5) trisphosphate; PTEN—phosphatase and tensin homolog; RAF—rapidly accelerated fibrosarcoma; RAS—rat sarcoma virus.

In contrast to the MAPK pathway, the PI3K/AKT pathway primarily regulates cell survival, metabolism, growth, and drug resistance [10,11]. Upon activation by EGFR or RAS, PI3K converts the membrane lipid PIP2 to PIP3, which recruits AKT to the plasma membrane, where it is phosphorylated and activated. Activated AKT promotes cell survival by inhibiting proapoptotic proteins and regulates numerous downstream targets involved in cell cycle progression, glucose metabolism, protein synthesis, and angiogenesis. The primary effector of AKT is the mammalian target of rapamycin (mTOR), which acts in two distinct complexes: mTORC1 and mTORC2. mTORC1 promotes protein synthesis, cell growth, and anabolic metabolism, whereas mTORC2 contributes to cytoskeletal organization and serves as an important activator of AKT, forming a positive signaling loop that enhances tumor survival and proliferation. A diagrammatic representation of EGFR signaling and resistance mechanisms to cetuximab and panitumumab is shown in Figure 2.

### 3.3. Pathway Cross-Talk and Inhibition

As discussed, the active RAS can directly activate both PI3K and MAPK pathways, proving *RAS* mutation hyperactivates both these pathways simultaneously. As such, inhibiting one pathway often leads cells to compensate by hyperactivation of the other pathway, hence, necessitating multi-target therapies.

## 4. Mechanisms of Resistance to Anti-EGFR Therapy

### 4.1. Primary Resistance

Resistance mechanisms can be broadly divided into genomic alterations (e.g., RAS, BRAF, and PIK3CA mutations), adaptive signaling processes (including feedback reactivation and receptor tyrosine kinase switching), microenvironment-mediated resistance, and novel epigenetic and immunological mechanisms. Epigenetic changes, including changes in DNA methylation, histone modifications, and regulation of noncoding RNA, can promote tumor plasticity and therapeutic adaptation without requiring the introduction of new genetic mutations. Similarly, immunological mechanisms, such as immune exclusion, immunosuppressive cytokine signaling, and interactions between tumor and stromal or immune cells, are increasingly recognized as contributing factors to therapeutic failure and resistance to targeted therapies.

KRAS, NRAS, and HRAS are three small GTPase proteins (p21) of the RAS family of proto-oncogene, which act as molecular switches regulating cell growth, differentiation, and survival. They cycle between active state (GTP bound) and inactive state (GDP bound) and work as downstream effectors within the mitogen-activated protein kinase (MAPK) pathway [12]. Mutations in this family represent a well-established intrinsic resistance mechanism to EGFR antagonists. Both intrinsic and acquired resistance mechanisms are associated with RAS. Preexisting RAS mutations driving EGFR-independent signaling are seen in intrinsic resistance, whereas the emergence of secondary KRAS mutations or resistant subclonal populations’ expansion during treatment is observed in acquired resistance [13]. Low-frequency subclonal RAS mutations, which can be missed by conventional sequencing, can also lead to intrinsic resistance, necessitating the need for highly sensitive methods like next-generation sequencing (NGS) and circulating tumor DNA (ctDNA) analysis to detect mutations and guide treatment strategies [14]. The detection of low-frequency RAS-mutant subclones remains analytically challenging, as conventional sequencing approaches may fail to identify variants present at low variant allele frequencies (VAFs) [15]. Modern NGS platforms and highly sensitive liquid biopsy assays can detect mutations at substantially lower frequencies, improving identification of clinically relevant resistant clones [16]. However, broader implementation of these technologies in routine clinical practice is limited by assay standardization, inter-laboratory variability, costs, turnaround times, and challenges in interpreting low-abundance variants, particularly when ctDNA shedding is limited. Dysregulation of this signaling axis is a crucial event in CRC carcinogenesis, with continuous activation of MAPK promoting tumor progression and growth [17]. Most of the KRAS mutations occur at codons 12 and 13 (exon 2); however, additional mutations in exons 3 and 4 of KRAS and exons 2 to 4 of NRAS are also identified, which are collectively termed as “expanded RAS mutation”. Mutations in KRAS exon 2 are common predictors of tumor resistance to EGFR antagonists [17]. Mutations in RAS, importantly, the KRAS-mutated tumors, are associated with around forty percent of CRCs [12,18,19], while NRAS is less frequent but remains a clinically relevant contributor to tumor heterogeneity. Mutations in RAS are a well-established negative predictive marker for EGFR antagonists, such as cetuximab and panitumumab. Such activating mutations in KRAS or NRAS result in impaired GTP hydrolysis, locking RAS in the active state and leading to continuous downstream signaling through the MAPK pathway, making EGFR inhibition ineffective. However, studies show that codon 13 mutated patients (G13D of KRAS gene/KRAS G13D) can confer partial sensitivity from cetuximab treatment compared to other mutated tumors, but prospective data remain inconclusive, and thus it is not clinically used to guide anti-EGFR therapy [17]. As such, the role of G13D mutation in primary resistance to EGFR antagonists remains controversial [17]. Overall, these mutations represent as critical predictive biomarkers of intrinsic resistance to EGFR antagonists and cetuximab or panitumumab should be avoided in patients exhibiting such mutations [17]. The result from PRIME trials shows that the “expanded RAS mutations” are predictive of resistance to anti-EGFRs [20]. It also showed that mutations located beyond the KRAS exon 2 can be a predictive factor for poor clinical outcome in combination therapy of panitumumab with first-line chemotherapy [17]. This study provided pivotal evidence supporting exclusion of patients with both KRAS and NRAS mutations from anti-EGFR therapy, defining the modern concept of “all RAS wild-type” selection for EGFR-targeted therapies. Combination therapy of KRAS inhibitor and anti-EGFRs, which is also referred to as the vertical inhibition, is now a validated strategy in molecularly selected subgroups to prevent such feedback.

BRAF is a serine/threonine protein kinase (signal transduction protein) and activating mutations in it accounts for around ten to fifteen percent of CRCs, mainly the BRAF V600E—a point mutation and a biomarker of poor prognosis [18,20,21,22]. It is involved in the regulation of cell differentiation, cell growth, and cell survival; as such, the BRAF mutation results in abnormal signaling pathways, leading to cell proliferation and inhibition of apoptosis by constitutively activating the MAPK pathway [17,23]. It is also associated with reduced responsiveness to EGFR antagonists and aggressive tumor biology. BRAF plays a crucial role in the MAPK pathway, and its constant activation bypasses the upstream EGFR signaling, leading to a low response compared to wild-type tumors [10,17]. It is considered as a negative predictive marker for EGFR monotherapy; however, its role as a predictive biomarker for therapy response is less definitive [24]. The BRAF mutation predicts resistance to EGRF antagonists, such as cetuximab and panitumumab, when used as monotherapy [17,21]. However, treatment strategies, such as the combination of cetuximab along with first-line chemotherapy, have shown that BRAF-mutated tumors gain additional benefits [17]. Additionally, BRAF and EGFR inhibitors combination therapy has shown an improved response rate in BRAF-mutated tumors.

### 4.2. Acquired Resistance

Acquired resistance develops after the initial period of clinical response. This resistance is driven by mechanisms such as ECD mutations (extracellular domains) and pathway bypasses (the kinome switch–HER2/MET amplification). In addition to intrinsic and acquired resistance, the neighborhood of the tumor plays a role. Such mechanisms include angiogenic escape and epithelial-to-mesenchymal transition (EMT) [25,26,27]. Resistance is rarely driven by a single mutation but rather by dynamic clonal selection under therapeutic pressure. International guidelines from various organizations and societies, such as the European Society of Clinical Oncology (ESMO), American Society of Clinical Oncology (ASCO), and National Comprehensive Cancer Network, suggest routine testing for mutations in RAS and BRAF, highlighting their importance [28,29,30,31,32]. Beyond bypass loops, mutations in the extracellular domain of EGFR (EGFR-ECD mutation), such as the S492R mutation, can change the receptor shapes, which in turn prevents cetuximab binding [33,34]. Fortunately, the S492R mutation does not confer resistance to panitumumab [33,35,36,37]. This mutation confers resistance to cetuximab but retains sensitivity to panitumumab due to distinct epitope binding [33,36]. The EGFR, HER2, MET, and IGF-1R are all RTKs that share the same downstream signal cascade (MAPK and PI3K), and resistance is essentially the tumor cells finding a different RTK to keep the signal cascade active. The dynamic nature of these resistance mechanisms, mainly the MET amplification and ECD mutations during treatment, highlights the clinical utility of longitudinal ctDNA monitoring to identify molecular relapses [33,38]. Despite strong preclinical evidence, clinical targeting of IGF-1R requires more clinical evidence to support its therapeutic application [39].

### 4.3. Bypass Signaling and Alternative Survival Pathways

#### 4.3.1. PI3K/AKT/mTOR Pathway Alterations

The phosphoinositide 3-kinase (PI3K)/AKT/mTOR pathway is a major downstream signaling cascade of EGFR and represents an important bypass mechanism contributing to resistance to cetuximab and panitumumab [40,41,42,43,44]. Activation of this pathway promotes cell survival, proliferation, metabolism, and therapeutic resistance independently of EGFR signaling. Although the PI3K family comprises three classes, resistance to anti-EGFR therapy is primarily associated with aberrant activation of Class I PI3Ks and downstream AKT/mTOR signaling [40,41,42,43,44,45].

PI3K is activated by receptor tyrosine kinases and RAS proteins, leading to the conversion of phosphatidylinositol 4,5-bisphosphate (PIP2) into phosphatidylinositol 3,4,5-trisphosphate (PIP3). This process recruits and activates AKT, which subsequently regulates multiple downstream effectors involved in cell survival, growth, and resistance to apoptosis [44,45]. Persistent activation of the PI3K/AKT/mTOR axis can, therefore, maintain proliferative signaling despite EGFR blockade.

Mutations in the PIK3CA gene, which encodes the p110α catalytic subunit of Class I PI3K, occur in approximately 15–20% of colorectal cancers and represent one of the most common alterations affecting this pathway [17,18]. The majority of mutations occur in hotspot regions within exons 9 and 20. Several studies have suggested that PIK3CA exon 20 mutations are associated with reduced sensitivity to anti-EGFR therapy, whereas the predictive significance of exon 9 mutations remains less consistent [17]. However, compared with RAS mutations, the role of PIK3CA alterations as independent predictive biomarkers of anti-EGFR resistance remains less clearly established.

Another important mechanism of PI3K pathway activation is loss of phosphatase and tensin homolog (PTEN), a tumor suppressor that negatively regulates PI3K signaling by dephosphorylating PIP3 to PIP2 [17]. PTEN loss, resulting from gene mutation, deletion, or epigenetic silencing, leads to constitutive activation of the PI3K/AKT pathway and has been associated with primary resistance to cetuximab and panitumumab. PTEN deficiency has been reported in approximately 20–40% of patients with mCRC; however, variability in assessment methods and the lack of standardized testing have limited its implementation as a routine predictive biomarker [10,17].

From a therapeutic perspective, activation of the PI3K/AKT/mTOR pathway has stimulated the development of PI3K, AKT, and mTOR inhibitors aimed at overcoming resistance to anti-EGFR therapy. While clinical results with single-agent pathway inhibitors have generally been modest, combination approaches involving anti-EGFR agents and PI3K pathway-targeted therapies continue to be investigated. These strategies may offer future opportunities to overcome resistance mediated by aberrant PI3K signaling and PTEN loss.

#### 4.3.2. HER2/HER3-Mediated Bypass Signaling

The ErbB family, consisting of EGFR (ERBB1), HER2 (ERBB2), HER3 (ERBB3), and HER4 (ERBB4), plays a crucial role in cell proliferation and cell survival regulation. Human epidermal growth factor receptor 2 (HER2/ERBB2) possesses potent kinase activity but lacks a known ligand, whereas HER3 has a ligand binding site but lacks a strong kinase activity [33,46]. HER2 is activated by heterodimerization with HER3 of the same family. HER3 has multiple binding sites/motifs for the PI3K (p85 regulatory subunit), making the HER2-HER3 heterodimer a powerful activator of intracellular signaling (PI3K/AKT survival pathway) [47]. HER2 amplification activates the MEK-ERK (MAPK) and PI3K-AKT pathways, rendering EGFR signal blockade irrelevant [10].

Consequently, patients with HER2 amplification exhibit poor response to anti-EGFR monotherapy. Studies reveal that patients harboring *RAS* wild-type mCRC with HER2-positive tumors showed worse PFS to anti-EGFR therapy [10]. HER2 amplification occurs in approximately 2–5% of all mCRC cases; however, its frequency can rise up to ten to fifteen percent in the *RAS* wild-type population [10]. Hence, HER2 amplification is a validated resistance biomarker and therapeutic target.

In HER2-amplified tumors, coexisting PIK3CA mutations may reduce the efficacy of HER2-targeted therapies, such as trastuzumab, by maintaining downstream PI3K/AKT signaling despite receptor inhibition [48]. This highlights the hierarchical nature of resistance, where a mutation in PI3K (downstream node) can negate the efficacy of EGFR/HER2 inhibitors (upstream receptors). Given the growing therapeutic importance of HER2-targeted strategies in mCRC, accurate assessment of HER2 status is becoming increasingly important. Current testing recommendations typically include initial immunohistochemistry (IHC) followed, in selected cases, by confirmatory in situ hybridization (ISH/FISH). HER2 positivity in mCRC is typically defined according to HERACLES-based criteria, which consider both staining intensity and the percentage of positive tumor cells. Standardized HER2 testing is particularly recommended for patients with RAS/BRAF wild-type tumors who develop resistance to anti-EGFR therapy, as HER2 amplification represents both a mechanism of resistance and a potential therapeutic target.

### 4.4. Tumor Microenvironment and Phenotypic Resistance

**Figure 3:**
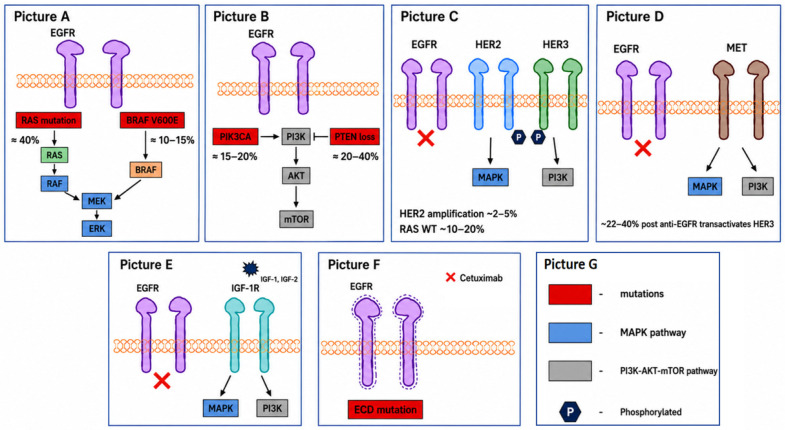
Schematic overview of the major mechanisms of resistance to anti-EGFR monoclonal antibodies (cetuximab and panitumumab) in metastatic colorectal cancer (mCRC). Therapeutic resistance arises through multiple genetic and microenvironmental alterations that bypass or reactivate the downstream signaling pathways. (**A**) MAPK pathway activation: *RAS* mutations (approximately 40% of mCRC) and *BRAF* mutations (*BRAF V600E*, approximately 10–15% of mCRC) constitutively activate the MAPK cascade (RAF-MEK-ERK) downstream signals, rendering anti-EGFR antibodies ineffective. (**B**) PI3K-AKT-mTOR pathway activation: *PIK3CA* mutations (approximately 15–20% of mCRC) and PTEN loss (approximately 20–40% of mCRC) lead to sustained PI3K-AKT-mTOR signaling, promoting cell survival and proliferation independently of upstream EGFR blockade. (**C**) HER2 bypass signaling: HER2 amplification (2–5% of mCRC) and HER2/HER3 heterodimerization activate both MAPK and PI3K pathways in RAS wild-type tumors (10–20% of resistance cases), independent of EGFR inhibition. (**D**) MET amplification and HER3 transactivation: MET amplification (22–40% of cases post-anti-EGFR therapy) transactivates HER3, reactivating downstream MAPK and PI3K signaling. (**E**) IGF-1R bypass signaling: activation of insulin-like growth factor 1 receptor (IGF-1R) by its ligands (IGF-1 and IGF-2) provides an alternative route to MAPK and PI3K activation. (**F**) EGFR ectodomain mutations: mutations in the extracellular domain of EGFR prevent binding of cetuximab and panitumumab while preserving receptor activation. (**G**) Tumor microenvironment: cancer-associated fibroblasts secrete ligands, promoting resistance through paracrine signaling.

Resistance is not always limited to the tumor cell itself—the tumor microenvironment (TME) also plays a significant role in therapeutic resistance. Nongenetic resistance mechanisms, mediated by the TME, are increasingly recognized as important contributors to therapeutic failure [2]. Growth factors, such as hepatocyte growth factor (HGF), a MET ligand, and neuregulin-1 (NRG1), a HER3 ligand, are secreted by cancer-associated fibroblasts (CAFs) within the TME. This paracrine signaling provides tumor cells with an alternative source of survival and proliferation signals, allowing them to maintain continued signaling despite effective EGFR blockade. Such mechanisms may explain therapeutic failure in a subset of tumors with wild-type RAS that lack identifiable intracellular resistance mutations [33]. Cancer-associated fibroblasts (CAFs) are one of the most abundant stromal cell populations in colorectal cancer and are increasingly recognized as key mediators of resistance to targeted therapies [49]. In addition to HGF and NRG1, CAFs secrete numerous cytokines and growth factors, including transforming growth factor β (TGF-β), interleukin 6 (IL-6), and CXCL12, which collectively promote tumor survival and progression [50]. By activating alternative receptor tyrosine kinases, such as MET and HER3, CAF-derived signals reactivate the MAPK and PI3K/AKT pathways despite EGFR inhibition. Consequently, cancer cells become less dependent on EGFR-dependent signaling and more capable of evading targeted therapeutic pressure. Another important mechanism of phenotypic resistance is epithelial-to-mesenchymal transition (EMT). EMT is characterized by the loss of epithelial markers, particularly E-cadherin, and the acquisition of mesenchymal characteristics [51]. This process is regulated by transcription factors that drive extensive cellular reprogramming. As tumor cells acquire a mesenchymal phenotype, they gain an increased capacity for migration and invasion, simultaneously becoming less dependent on EGFR signaling. EMT is, therefore, associated with reduced sensitivity to anti-EGFR therapies and poorer clinical outcomes in metastatic colorectal cancer. The immune compartment of the tumor microenvironment also contributes to treatment resistance. Tumor-associated macrophages (TAMs) [52], regulatory T cells (Tregs), and myeloid-derived suppressor cells (MDSCs) create an immunosuppressive environment that promotes tumor progression and limits the antitumor immune response [53]. In addition, inflammatory cytokines activate intracellular signaling pathways, promoting cell survival, proliferation, and resistance to apoptosis. Persistent inflammatory signaling may, therefore, facilitate the emergence and expansion of resistant tumor cell populations. Hypoxia constitutes another important element of the resistant tumor microenvironment. As tumors grow beyond the reach of blood vessels, hypoxia leads to the stabilization of hypoxia-inducible factor 1α (HIF-1α), which then induces the expression of vascular endothelial growth factor (VEGF) and promotes angiogenesis [54]. Hypoxia also drives metabolic adaptation, genomic instability, and EMT, further contributing to resistance to anti-EGFR therapies. These adaptations allow tumor cells to survive under stress conditions and reduce their dependence on EGFR signaling [55]. The gut microbiome may further influence the therapeutic response in colorectal cancer. Specific bacterial species have been associated with colorectal cancer progression and may promote resistance by activating inflammatory pathways, modulating immune responses, and improving tumor cell survival. Although the clinical significance of microbiome-mediated resistance is still under investigation, this area represents a promising avenue for future therapeutic interventions. Emerging evidence also suggests that epigenetic reprogramming and immune-mediated mechanisms may contribute to resistance to anti-EGFR therapies. Alterations in DNA methylation, histone modifications, and microRNA expression may promote tumor plasticity, epithelial-to-mesenchymal transition, and adaptive drug tolerance. Concurrently, an immunosuppressive tumor microenvironment characterized by regulatory immune cells, myeloid-derived suppressor cells, and cytokine-dependent signaling may reduce therapeutic efficacy and facilitate tumor survival. Although these mechanisms are less well understood than canonical genomic resistance factors, they represent important areas of ongoing research and potential therapeutic intervention. Figure 3 shows a summary of the main mechanisms of resistance to anti-EGFR monoclonal antibodies in metastatic colorectal cancer (mCRC).

### 4.5. Clinically Effective Resistance Mechanisms

The growing understanding of resistance mechanisms has enabled the development of biomarker-based therapeutic strategies for metastatic colorectal cancer. Several molecular alterations, once considered solely resistance mechanisms, have now become effective therapeutic targets. Advances in molecular profiling, liquid biopsy technologies, and the development of targeted drugs have facilitated the implementation of precision oncology approaches aimed at overcoming resistance and improving treatment outcomes. Consequently, modern treatment algorithms increasingly rely on the identification of specific genomic alterations, which form the basis for therapeutic decision-making. Table 1 summarizes clinical studies evaluating targeted therapy and biomarker-guided therapies in mCRC. It includes treatments directed against BRAF V600E mutations, KRAS G12C mutations, HER2 amplification, MET amplification, and PIK3CA mutations. It also includes studies investigating anti-EGFR therapy selection and rechallenge strategies guided by ctDNA. Trial name, patient population, biomarker selection, treatment regimen, trial phase, and main outcomes are presented.

Among the effective resistance mechanisms, BRAF V600E mutations are one of the most clinically validated targets. The phase III BEACON CRC trial demonstrated improved overall survival with encorafenib plus cetuximab compared to standard chemotherapy [56]. Recently, the BREAKWATER trial demonstrated significant improvements in progression-free survival and overall survival after combining encorafenib and cetuximab with first-line chemotherapy, establishing a new treatment paradigm for patients with BRAF-mutant mCRC [57].

HER2 amplification has emerged as both a resistance mechanism and a therapeutic target. Clinical trials such as HERACLES [60], MyPathway [61], DESTINY-CRC01 [59], and MOUNTAINEER [62] have demonstrated significant clinical activity of HER2-targeted therapies in patients with HER2-positive, RAS-wild-type mCRC. These findings support routine HER2 testing in appropriate patient populations and underscore the role of dual HER2 blockade and antibody–drug conjugates in overcoming resistance to anti-EGFR therapy. Historically, KRAS mutations have been considered untreated therapeutic targets. However, the development of KRAS G12C inhibitors has significantly changed the therapeutic landscape. The phase III CodeBreaK 300 trial demonstrated improved progression-free survival with the combination of sotorasib and panitumumab compared with standard treatment, supporting the concept of vertical pathway inhibition in KRAS G12C-mutated tumors [58]. MET amplification represents a clinically relevant mechanism of acquired resistance. Early clinical trials suggest that combined MET and EGFR inhibition can restore treatment sensitivity in selected patients. Ongoing studies are aimed at determining the optimal role of MET-targeted therapies in overcoming resistance to anti-EGFR drugs [70]. Liquid-biopsy-based monitoring has further expanded precision oncology methods. Serial ctDNA analyses enable the detection of emerging resistance mutations and the identification of patients who may benefit from anti-EGFR retreatment strategies. Studies such as CRICKET and CAVE have demonstrated that patients with elimination of resistant RAS and EGFR mutant clones can regain sensitivity to cetuximab-based therapy, reinforcing the need to integrate molecular monitoring into clinical decision-making [68,69]. Collectively, these advances illustrate the shift from empirical treatments to biomarker-driven precision medicine. Integrating genomic profiling, ctDNA monitoring, and targeted therapeutic combinations offers new opportunities to overcome resistance and improve long-term outcomes in metastatic colorectal cancer.

## 5. Strategies to Overcome Resistance to Anti-EGFR Therapy

The emergence of previously discussed resistance mechanisms to anti-EGFR antibodies, including cetuximab and panitumumab, has necessitated the development of rational therapeutic strategies aimed at restoring pathway control. Such approaches are mainly based on targeting downstream effectors, inhibiting parallel signaling, and adaptive treatment guided by tumor evolution. A comprehensive understanding of resistance biology enabled the transition from monotherapy to biomarker-driven combination regimens.

### 5.1. Vertical Pathway Inhibition

As discussed the vertical inhibition refers to simultaneous targeting of multiple nodes within the same signaling cascade. Such an example is the EGFR-RAS-RAF-MEK-ERK axis. This approach can help to overcome feedback activation.

In *BRAF*-mutated CRC, use of the *BRAF* inhibitor as monotherapy has demonstrated limited clinical outcome due to rapid reactivation of the EGFR signal cascade, distinguishing it from melanoma. Therapeutically, targeting *BRAF* mutant CRCs has evolved significantly in recent years. The BEACON CRC trial led to a combination of encorafenib (*BRAF* inhibitor) and cetuximab as a standard care of *BRAF V600E* mCRC, showcasing improvements in overall survival and response rates compared to conventional chemotherapy [56]. More resent trials, such as the phase III BREAKWATER trial (2024–2025), demonstrated that combination strategy of (a) encorafenib and cetuximab along with (b) chemotherapy, such as mFOLFOX6, has greatly improved the response rate and survival outcomes, supporting a shift toward biomarker-driven combination strategies in the first-line setting [10,73,74]. Such findings highlight the role and importance of dual pathway inhibition. However, challenges remain despite such advances.

Similarly, *KRAS* G12C mutations can be targeted using sotorasib (*KRAS* inhibitor); however, such monotherapy can lead to adaptive feedback activation of the EGFR cascade. Adaptive feedback activation of EGFR limits the efficacy of KRAS G12C inhibitors, thereby necessitating combination strategies. Interestingly, KRAS G12C (glycine to cysteine) can be targeted using the covalent inhibitors, such as sotorasib, in combination with EGFR antagonists, such as panitumumab [75]. This combination strategy counteracts the feedback activation of the EGFR-RAS-MAPK axis and enhances pathway suppression. The clinical trial CodeBreaK 300 phase III, which focused on KRAS G12C, showed that the combination of sotorasib and panitumumab significantly improved the progression-free survival (PFS) compared to standard chemotherapy [58]. Although improved, responses remain limited, highlighting adaptive resistance.

### 5.2. Horizontal Pathway Inhibition and Bypass Pathway Blockade

This strategy focuses on targeting parallel signaling pathways, which allows the tumor to bypass EGFR inhibition. Amplification of RTKs, such as HER2 and MET, are well-known mechanisms of resistance in mCRC [76].

As discussed earlier, HER2 amplification leads to activation of both the MAPK and PI3K/AKT signaling pathways, independent of EGFR. In response to such challenges, clinical trials, such as DESTINY-CRC01, HERACLES, MyPathway study, MOUNTAINEER, and TRIUMPH, explored targeted therapies against HER2 in mCRC [59,60,61,62,63,77,78,79]. The DESTINY-CRC01 trial investigated an antibody–drug conjugate (ADC), namely, trastuzumab deruxtecan (T-Dxd), which delivers a payload of chemotherapy directly to HER2-expressing cells [77,78]. Remaining trials evaluated trastuzumab with lapatinib/pertuzumab/tucatinib, which also showed responses to HER2-positive mCRC [33,80]. The HERACLES and MyPathway trials evaluated dual HER2 blockade (trastuzumab in combination with lapatinib or pertuzumab) and the MOUNTAINEER trial assessed trastuzumab in combination with tucatinib, which contributed to the establishment of HER2-targeted therapy as a treatment option in refractory settings [60,61,62]. The TRIUMPH phase II (EPOC1602) trial focused on the combination of trastuzumab and pertuzumab in *RAS* wild-type HER2-positive mCRC [10].

The TRIUMP and DRUP trials further support the efficacy of dual HER2 inhibition in RAS wild-type, HER2-positive mCRC. The CRYSTAL trial highlighted that the combination of cetuximab with FOLFIRI improved PFS and overall response rate [33]. Likewise, the PARADIGM trial (combination of panitumumab and mFOLFOX6) in patients with *RAS* wild-type, left-sided mCRC showed increased overall survival [33]. The VALENTINO and PANAMA trials showed that after an initial course of panitumumab + FOLFOX, discontinuing oxliplatin and continuing with panitumumab with fluoropyrimidines, such as fluorouracil/leucovorin, may help in maintaining disease control while lowering its toxicity. The idea of rechallenging is becoming more important in the treatment of cancer. By using ctDNA, it is possible to check if resistance-related mutations, such as *RAS* or EGFR, have disappeared after ceasing the treatment. If such mutations are not detected, it suggests the tumor may become sensitive to anti-EGFR therapy. Studies such as CRICKET and CAVE showed that restarting the same therapy in carefully selected patients can lead to increased overall survival and ORR [33].

Collectively, these studies establish HER2 amplification as both a mechanism of resistance and a therapeutic target in mCRC.

Similarly, MET amplification, especially in the acquired resistance setting, can drive tumor progression despite EGFR blockade. Since MET acts as a bypass, combination therapy with a MET inhibitor can resensitize the tumor to EGFR therapy. Such an example is the PERSPECTIVE study—a clinical trial investigating a combination of a MET inhibitor (tepotinib/savolitinib) with EGFR antagonists (cetuximab) to shut down both main signaling pathways and bypass simultaneously [70].

These findings suggest that co-targeting multiple receptor pathways is necessary to overcome signaling redundancy and restore the therapeutic response. However, despite such advances, the response rate remains limited, and resistance eventually develops, highlighting the importance of combination strategies and improved biomarker-driven patient selection.

### 5.3. Combined EGFR and Angiogenesis Inhibition

Angiogenesis is an important factor in tumor progression and resistance. The combination of bevacizumab (anti-angiogenic agents) and EGFR inhibitors has been explored as a strategy to improve the therapeutic outcome [81,82]. However, few clinical trials suggested an improved response rate, while others showed increased toxicity without significant survival benefit. This outcome indicates that patient selection and treatment sequencing are crucial when using such combination therapies.

### 5.4. Targeting the PI3K/AKT/mTOR

Alterations in the PI3K/AKT signaling pathway, including PIK3CA mutation and PTEN loss, greatly contribute to tumor resistance by maintaining downstream signaling independent of EGFR blockade. Targeting this pathway using their inhibitors is investigated. However, monotherapy has shown minimal clinical benefit. To overcome such limitations, multiple combination strategies have been explored.

A combination of alpelisib (oral selective inhibitor of class I PI3K p110α) and capecitabine, an oral prodrug of 5-FU (fluorouracil), in a Phase Ib trial showed that in PIK3CA mutant CRC, the combination therapy was tolerable and showed antitumor activity [71]. One of the most unique combination approaches is the use of aspirin alongside standard therapy. Patients with a PIK3CA mutation showed significant survival benefit from regular aspirin use [72].

## 6. Role of Liquid Biopsy, ctDNA, and NGS

**Figure 4:**
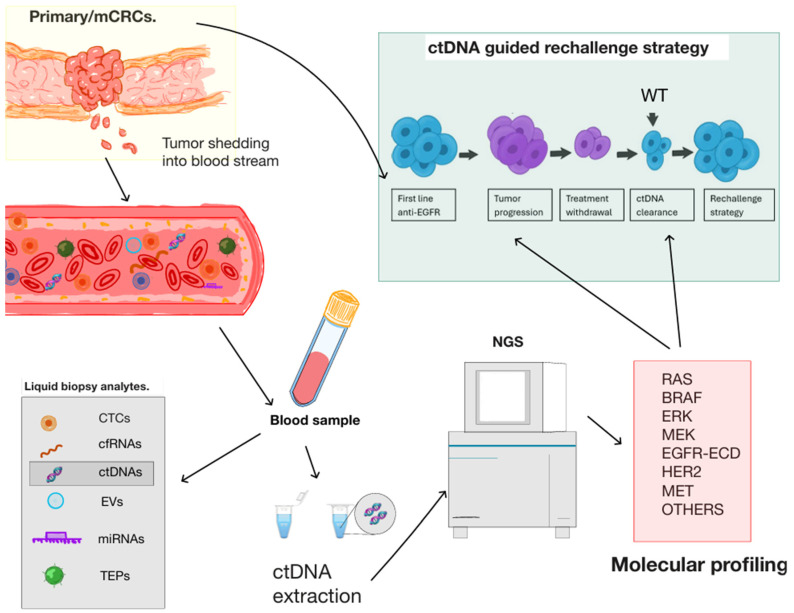
Treatment strategy for patients with metastatic colorectal cancer (mCRC) based on liquid biopsy. This schematic diagram illustrates the liquid-biopsy-driven rechallenge strategy for metastatic colorectal cancers (mCRCs). Following first-line anti-EGFR therapy, tumor progression leads to the shedding of tumor components, such as circulating tumor DNA (ctDNA), into the bloodstream. A blood sample is collected, from which various liquid biopsy analytes (such as CTCs, cfRNAs, ctDNAs, EVs, miRNAs, and TEPs) are extracted. Molecular profiling via next-generation sequencing (NGS) identifies alterations in key genes (e.g., RAS, BRAF, ERK, MEK, EGFR-ECD, HER2, MET, and others). Upon treatment withdrawal, ctDNA clearance is observed, enabling a rechallenge strategy with anti-EGFR therapy when the tumor returns to a wild-type (WT) status, as guided by longitudinal ctDNA monitoring. Abbreviations: anti-EGFR—anti-epidermal growth factor receptor; CTCs—circulating tumor cells; cfRNAs—cell-free RNAs; EVs—extracellular vesicles; miRNAs—microRNAs; TEPs—tumor educated platelets; NGS—next-generation sequencing; WT—wild-type; RAS—rat sarcoma viral oncogene; BRAF—v-RAF murine sarcoma viral oncogene homolog B1; ERK—extracellular signal-regulated kinase; MEK—mitogen-activated protein kinase kinase; EGFR-ECD—epidermal growth factor receptor extracellular domain; HER2—human epidermal growth factor receptor 2; MET—mesenchymal-to-epithelial transition factor.

In the management of mCRC, the use of liquid biopsy by analyzing ctDNA via NGS (Figure 4) has become a transformative tool for overcoming both intrinsic and acquired tumor resistance to EGFR antagonists, such as panitumumab and cetuximab. Analytes, such as the ctDNA, extracellular vesicles (EVs), cell-free RNAs (cfRNAs), circulating tumor cells (CTCs), tumor-associated microRNAs, and tumor-educated platelets, can be assessed using liquid biopsy [83]. Unlike traditional biopsies, which are invasive and pose risks, ctDNA provides a real-time view of the tumor’s evolution at the molecular level and treatment response [83]. Additionally, clinical trials prove that treatment based on molecular profiling shows a significant increased response rate and overall survival. Negative hyperselection is a strategy used in patient selection for anti-EGFR therapy by expanding molecular profiling beyond *RAS* and *BRAF* standard evaluation [10]. This is critical for identifying secondary mutations in the MAPK pathway, pathway bypass mechanisms that often drive resistance.

ctDNA is a baseline stratification in anti-EGFR treatments and a minimally invasive liquid biopsy where small DNA fragments shed by tumors into body fluids, mainly the bloodstream, are used for genomic profiling via NGS (molecular sequencing) [10]. It helps in monitoring tumor response, resistance, new resistance mechanisms, as well as guiding rechallenge strategies and predicting relapse. However, there are several limitations, such as standardization, assay performance, economic feasibility, limited tumor shedding, restricted access to the bloodstream, and false negatives due to low shedding [10,83,84].

## 7. Conclusions and Future Perspectives

Resistance to cetuximab and panitumumab (anti-EGFR therapies) remains a major hurdle in management of mCRC. Both intrinsic and acquired resistance coverage on reactivation of downstream signaling pathways, bypass signaling, and nongenetic factors contribute to therapeutic failure. Advances in molecular profiling have greatly improved the understanding of tumor heterogeneity and clonal evolution, which has led to development of rational therapeutic strategies, such as vertical pathway inhibition, dual receptor blockade, and novel combination therapies. The integration of liquid biopsy, ctDNA, and NGS into clinical practice represents a step toward personalized treatment adaption. These approaches enable early detection of resistance mechanisms and facilitate treatment strategies, such as rechallenging. However, responses remain limited, highlighting the need for continued research into resistance biology, biomarker selection, and novel combination strategies. Ultimately, overcoming resistance to therapy requires a comprehensive, precise, and adaptive oncological approach that integrates genomic, molecular, and clinical data for better treatment outcomes. Despite significant progress in understanding resistance to anti-EGFR therapies in metastatic colorectal cancer, several challenges remain. Future research should focus on developing combination treatment strategies that can simultaneously target multiple resistance pathways. In particular, combining anti-EGFR drugs with immunotherapeutic approaches may help overcome resistance mediated by the tumor microenvironment and increase treatment efficacy in selected patient populations. Another promising area is the use of artificial intelligence and machine learning techniques to integrate genomic, transcriptomic, proteomic, and clinical data to improve biomarker discovery and patient stratification. Such approaches may facilitate the identification of novel resistance mechanisms and enable more personalized treatment decisions.

New technologies for monitoring resistance are also expected to play an increasingly important role. Analyses of serial circulating tumor DNA, single-cell sequencing, spatial transcriptomics, and multiomic profiling offer opportunities for earlier detection of resistant clones and real-time assessment of tumor evolution. Integrating these technologies into clinical practice could improve therapy selection, optimize rechallenge strategies, and ultimately improve outcomes for patients receiving anti-EGFR therapy.

## Figures and Tables

**Table 1:** Selected biomarker-guided clinical trials in metastatic colorectal cancer (mCRC): patient selection strategies, therapeutic regimens, and key clinical outcomes.

Category	Trial Name	Patient Population	Biomarker Selection	Treatment Regimen	Trial Phase	Main Outcomes	References
Experimental (*BRAF V600E* combination)	BEACON CRC	*BRAF V600E* mutant mCRC (previously treated)	*BRAF V600E*	Encorafenib + cetuximab ± binimetinib	Phase III	Improved OS vs. chemo	[56]
Experimental (*BRAF V600E* combination)	BREAKWATER (2024–2025)	*BRAF V600E* mCRC (first-line)	*BRAF V600E*	Encorafenib + cetuximab + mFOLFOX6	Phase III	Improved OS and PFS vs. chemo alone	[57]
Experimental (*KRAS G12C* inhibition)	CodeBreaK 300	*KRAS G12C* mutant mCRC	*KRAS G12C*	Sotorasib + panitumumab	Phase III	Improved PFS vs. standard chemo	[58]
Experimental (HER2-targeted therapy)	DESTINY-CRC01	*HER2*-expressing mCRC (IHC 3+ or 2+/ISH+)	*HER2* amplification	Trastuzumab deruxtecan (T-DXd)	Phase II	ORR ~45% in IHC 3+	[59]
Experimental (HER2-targeted therapy)	HERACLES	RAS wild-type, HER2+ mCRC	*HER2* amplification	Trastuzumab + lapatinib	Phase II	ORR ~30% responses	[60]
Experimental (HER2-targeted therapy)	MyPathway	HER2-amplified mCRC	*HER2* amplification	Trastuzumab + pertuzumab	Phase IIa	ORR 32%	[61]
Experimental (HER2-targeted therapy)	MOUNTAINEER	RAS wild-type, HER2+ mCRC	*HER2* amplification	Trastuzumab + tucatinib	Phase II	ORR 38.1%	[62]
Experimental (HER2-targeted therapy)	TRIUMPH (EPOC1602)	RAS wild-type, HER2+ mCRC	*HER2* amplification	Trastuzumab + pertuzumab	Phase II	Confirmed efficacy	[63]
Clinically established/ standard-of-care (combination with chemotherapy)	CRYSTAL	RAS wild-type mCRC	Prospective enrollment with retrospective RAS analysis	Cetuximab + FOLFIRI	Phase III	Reduced the risk of progression in RAS wild-type	[64]
Clinically established/ standard-of-care (combination with chemotherapy)	PARADIGM	RAS wild-type, left-sided mCRC	RAS wild-type + Left-sided primary	Panitumumab + mFOLFOX6	Phase III	Increased OS vs. bevacizumab + chemo	[65]
Clinically established/ standard-of-care (Maintenance therapy)	VALENTINO/PANAMA	RAS wild-type mCRC (after induction)	RAS wild-type	Panitumumab + fluoropyrimidine (5-fluorouracil/leucovorin) after discontinuing oxaliplatin	Phase II	Maintained disease control, reduced toxicity	[66,67]
Experimental (ctDNA-guided rechallenge)	CRICKET/CAVE	Patients with undetectable *RAS/EGFR* mutations in ctDNA after treatment break	ctDNA-guided (RAS/EGFR mutation clearance)	Anti-EGFR rechallenge	Phase II	Improved OS and ORR in ctDNA-selected patients	[68,69]
Experimental (MET amplification targeting)	PERSPECTIVE study	MET-amplified, anti-EGFR-resistant mCRC	*MET* amplification	MET inhibitor (tepotinib/savolitinib) + cetuximab	Phase I/II	Resensitization to EGFR blockade	[70]
Experimental (PI3K/AKT pathway targeting)	Phase Ib trial	*PIK3CA* mutant mCRC	*PIK3CA* mutation	Alpelisib + capecitabine	Phase Ib	Tolerable; antitumor activity observed	[71]
Observational	Observational/cohort	*PIK3CA* mutant mCRC	*PIK3CA* mutation	Aspirin + standard therapy	Not a trial (cohort study)	PIK3CA-associated adverse prognosis was not observed among aspirin users, although no significant interaction was detected	[72]

Abbreviations: ctDNA—circulating tumor DNA; EGFR—epidermal growth factor receptor; HER2—human epidermal growth factor receptor 2; IHC—immunohistochemistry; ISH—in situ hybridization; mCRC—metastatic colorectal cancer; ORR—objective response rate; OS—overall survival; PFS—progression-free survival;.

## Data Availability

No new data were created or analyzed in this study. Data sharing is not applicable to this article.
